# Simulations of the 2D self-assembly of tripod-shaped building blocks

**DOI:** 10.3762/bjnano.11.73

**Published:** 2020-06-08

**Authors:** Łukasz Baran, Wojciech Rżysko, Edyta Słyk

**Affiliations:** 1 Department for Theoretical Chemistry, Institute of Chemical Sciences, Faculty of Chemistry, Maria Curie-Sklodowska University in Lublin, Poland.

**Keywords:** 2D materials, coarse-grained model, molecular simulations, self-assembly, structural characterization, tripod building blocks

## Abstract

We introduce a molecular dynamics (MD) coarse-grained model for the description of tripod building blocks. This model has been used by us already for linear, V-shape, and tetratopic molecules. We wanted to further extend its possibilities to trifunctional molecules to prove its versatility. For the chosen systems we have also compared the MD results with Monte Carlo results on a triangular lattice. We have shown that the constraints present in the latter method can enforce the formation of completely different structures, not reproducible with off-lattice simulations. In addition to that, we have characterized the obtained structures regarding various parameters such as theoretical diffraction pattern and average association number.

## Introduction

On-surface synthesis is a newly developing field in chemistry that aims at making use of solid surfaces as a confinement template to initiate chemical reactions. It can be thought of as an extension of heterogenous catalysis where the initial precursors, the intermediate state, and the final supramolecular network all remain in an adsorbed state. Complex self-assembled structures are essential for many problems in the chemical industry such as gas storage, chemical sensing, and drug delivery [1–3]. Thus, this field has very recently gained particular interest in both experimental and theoretical studies, which was followed by a vast amount of papers devoted to investigating these phenomena. Thanks to this research, several factors have been established that can help to control the self-assembly process, such as precursor design [4–5], substrate nature and symmetry [6–7], type of solvent and its concentration, and thermodynamic conditions. The knowledge of the influence of these variables is crucial to reduce time, cost, and effort regarding the preparation of networks with predefined structural properties.

There are various molecules that possess the ability to self-assemble on solid surfaces. One of the most interesting types are building blocks of linear [8–12], V-shape [13–14], tripod [15–16], tetrapod [17–21], and hexapod [22] architecture. Also, a lot of effort has been put into the investigation of the mixtures [23–25] and the guest-induced self-assembly [26] of aforementioned particles. An important aspect of the on-surface synthesis is that a lot of these chemical reactions are impossible to be performed in bulk. This is mainly because the substrate reduces the number of rotational and translational degrees of freedom of the admolecules. As a consequence, intermolecular contacts occur that stabilize the interactions between the particles.

To investigate such phenomena in more detail, a proper methodology for the development of complex structures is of particular interest. However, the properties of these structures are hard to predict due to the high number of possible parameters that influence their formation. Thus, it is necessary to use computer modeling, which allows for a versatile examination of various thermodynamic conditions in acceptable time frames. Additionally, it is also a convenient tool to vary multiple factors such as the shape of the molecules, and the type of solvent and substrate. The insight gained from the simulations can lead to valuable conclusions, which can be further explored and proved by experimental studies.

To date, there are two main approaches that can complement the results obtained in experiments. The first one involves the use of all-atom simulations by molecular dynamics (MD) [27–30]. Even though these models are able to compare explicitly the quantities measured in experiments, the possibilities in the prediction of structural properties are limited due to the complex form of the employed interparticle potentials used in the empirical force field, such as Amber99sb [31–32] or MMFF94 [33]. It follows that it is only possible to investigate tens of molecules in total in a reasonable time. The second approach involves the use of simple coarse-grained models, which have been shown to reflect already existing experimental data and to predict new structures, which have not been observed yet. Simulations for this kind of models have been performed using lattice [34–35] and off-lattice [36–38] Monte Carlo simulations, or MD simulations [37,39]. Regarding the latter, we have very recently shown that this methodology is suitable for the representation of tetratopic molecules with different directions of interparticle interactions [37,39], as well as for linear, V-shape, and tetraphenylethylene derivatives [40].

In this work, we want to show that the possibilities of our MD coarse-grained model are not limited to these geometries but can also be used for tripod molecules. In addition to that, we have also compared the results with Monte Carlo results on a triangular lattice (l-MC). We have shown that not for every system the results obtained from both methods agree. Obviously, the latter method is not always an adequate tool for the investigation of molecules of this type, due to the constraints present on the lattice, which might enforce the formation of structures congruent with the lattice symmetry. Moreover, we have characterized the obtained structures regarding various structural parameters such as structure factor and distribution of association number.

The outline of this paper is as follows. In the next section, we describe our model and simulation details used in the course of our study. Then, we present the results of our simulations, which show various structures, depending on the molecular architecture, and their characterization. Finally, we briefly summarise our findings.

## Model and Simulation Details

**Figure 1:**
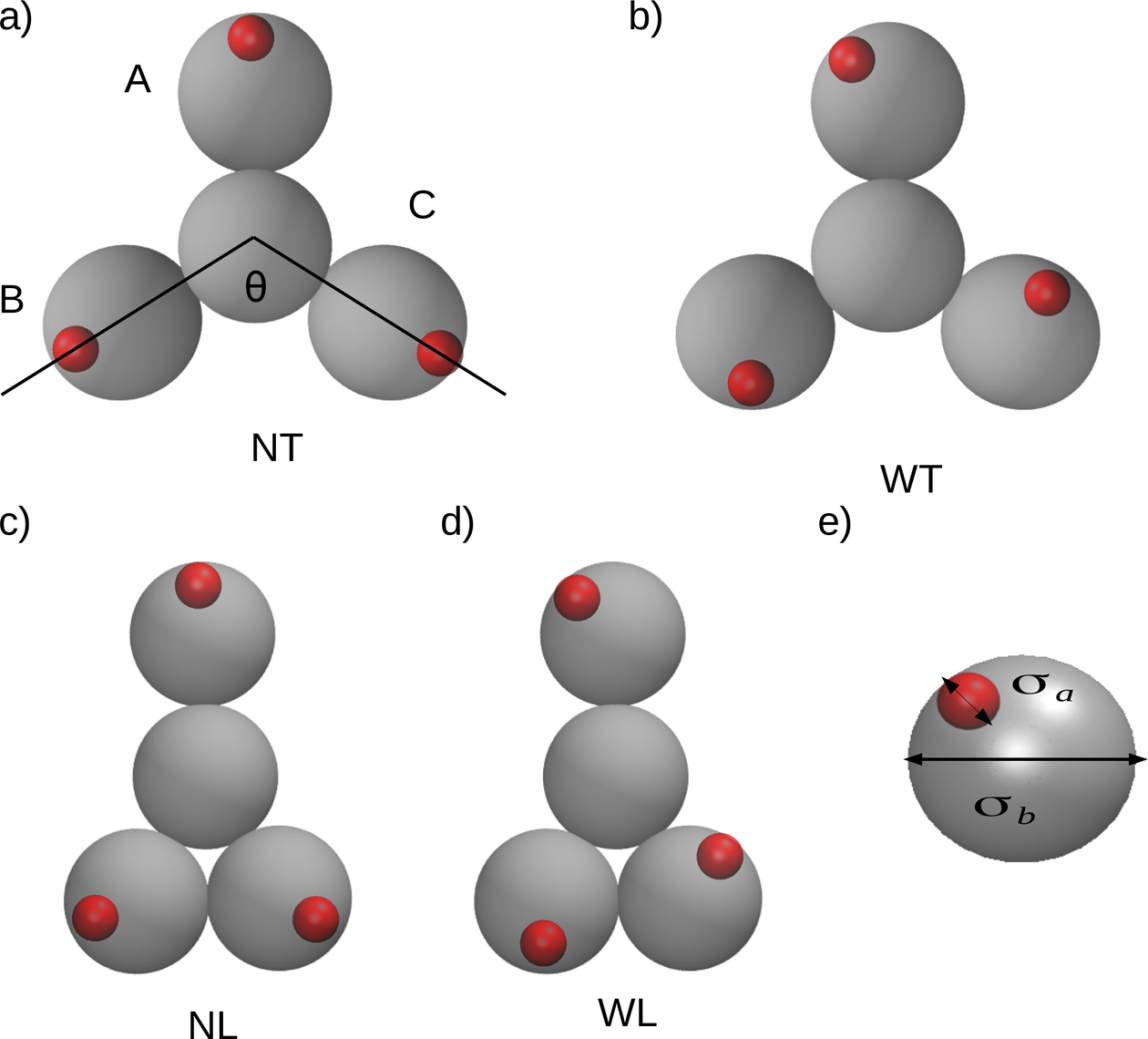
a–d) Schematic representation of the models used in this work. Silver and red circles correspond to the components of the backbone and “phantom” active sites, respectively. In a) we can see that the arms are marked as A, B, and C. e) Parameters of the molecular dynamics model.

In this paper, we have used the same approach as in [37,39–40], which is a coarse-grained MD model, now extended to describe the behavior of tripod building blocks. One of the examples of chemical compounds with this molecular geometry is benzene-1,3,5-tricarboxylic acid, more commonly known as trimesic acid. In our investigations, every molecule was treated as a flat and rigid object. The molecules were modeled with one center segment to which three arms are attached. The beads are of equal size σ*_b_*, so we will not distinguish them, but rather refer to them as the components of the entire backbone. The length of each of the arms has been changed in the course of the simulations to investigate also asymmetrical molecules. The three lengths of the arms are denominated **A**, **B**, and **C** as shown in Figure 1a. From the chemical point of view, the length of the arms and the chemical nature of the “active” groups can be controlled by the use of different substituents, for instance, a different number of connected phenyl groups [18–19]. To simplify the notation, we will refer to every model as **MABC**, where **M** means the name of the model and **A**, **B**, and **C** are the length of each arm. As shown in Figure 1a, we have marked the angle θ between arms **B** and **C**. This angle has been set for the models **NT** and **WT** to θ =120°and to θ = 60°for the models **NL** and **WL**. One has to note that for the latter models we can not reproduce the counterparts in the Monte Carlo simulations on a triangular lattice. The active sites, each of size σ*_a_*, which are supposed to reflect directional interactions are grafted onto the terminal segment of each arm and the bonding distance between them is abbreviated as *l*.

To ensure the distances between particular beads and active sites in the MD simulations we have used the harmonic binding potentials

[1]
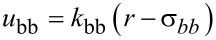


and

[2]
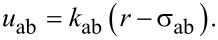


The same approach has been used to maintain small fluctuations of the angles:

[3]
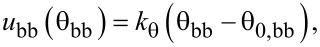


[4]
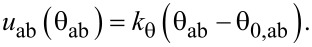


The interparticle potential used was the Lennard-Jones 12,6 potential, which was shifted in such way that potential and forces are continuous at the cut-off distance [41]:

[5]
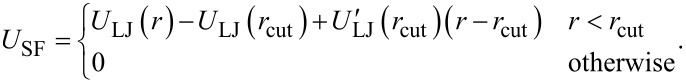


where *U*_LJ_(*r*) = 4ε*_kl_*[(σ*_kl_*/*r*)^12^ − (σ*_kl_*/*r*)^6^], and *U*′_LJ_(*r*_cut_) is the first derivative of *U*_LJ_(*r*) at *r* = *r*_cut_. The backbone Lennard-Jones parameters σ = σ_b_ and ε = ε_bb_ have been set to be the units of length and energy, respectively. Reduced temperature and timestep have been defined as T* = *kT*/ε and 
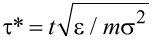
, respectively. The number density is equal to


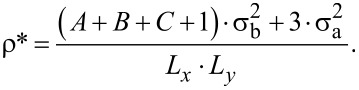


The mass of backbone constituents as well as of the active sites were set to unity, their diameters were set to σ*_b_* = 1.0 and σ*_a_* = 0.2. The distance between active site and the terminal segment of the arm segment was set to *l* = 0.5, which ensures that three tripod molecules can associate simultaneously. The energies of the backbone–backbone and backbone–site interactions were set to ε_bb_ = ε_ab_ = 1.0ε, while ε*_aa_* = 4.0ε. Briefly, these parameters were chosen so that no other interactions were taken into account, except the highly directional interparticle interactions, which can mimic, for instance, the association of carboxylic acid groups.

The diameters of pairs were calculated from the mixing rule σ*_kl_* = (σ*_k_* + σ*_l_*)/2, where *k*, *l* = a ,b. The cut-off distance for active sites were set to *r*_cut,aa_ = 2.5σ*_aa_*, while the remaining were set to *r*_cut,ij_ = σ*_ij_*, where *i*, *j* = *ab*, *bb*. The latter means that the only attraction in the system is due to the interactions between active sites. The harmonic potential constants are equal to *k*_bb_ ≡ *k*_ab_ = 1000ε/σ^2^ and *k*_θ_ = 1000ε/rad^2^.

All MD simulations have been performed in the *NVT* ensemble with the LAMMPS simulation package [42–43]. The standard velocity-Verlet integration scheme has been used with a reduced timestep of t* = 0.002τ. To maintain a constant temperature, the system has been preliminarily equilibrated with a Berendsen thermostat for 5·10^6^ simulations steps and after that, we have switched to the Nosé–Hoover chains scheme for further equilibration for at least 5·10^7^ simulation steps. The parameters of the latter thermostat were as follows: number of chains *N*_chain_ = 3 and dampening constant τ_NH_ =10τ. The system was slowly cooled down from disordered systems to the point where self-assembled structures appeared using a temperature grid of ΔT* = 0.01.

In the Monte Carlo simulations, we have assumed that one segment can occupy only one vertex of a triangular lattice and the interaction energy between terminal arm segments is taken into account only if the neighboring arms are collinear (→←), resulting in ε = −1 energy contribution. One has to take into account that in this method we do not need smaller active sites as in the case of MD. To explicitly compare temperatures from both methods, we have multiplied the temperatures of lattice systems by ε_aa_, and abbreviated it as T*_l−MC_. The total amount of molecules was set to 2500 for both methods. However, it is important to highlight that the total number of atoms were different depending on the tripod geometry.

## Results and Discussion

**Figure 2:**
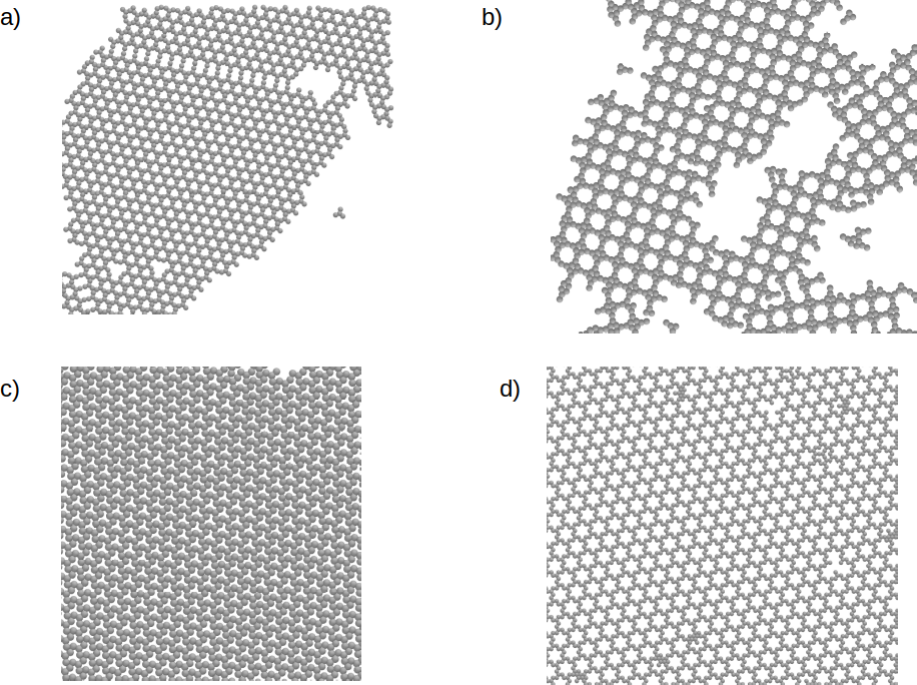
Part of the configurations for a) **NT111** at T* = 0.58, b) **NL111** at T* = 0.54, and c) **WT111** at T* = 0.58, all with ρ* = 0.2. d) Corresponding Monte Carlo simulation on a triangular lattice for **WT111** at T*_l−MC_ = 1.2.

We start our discussion with the presentation of the results for the models **M111**, in which every arm consists of only one bead. Parts of the configurations are shown in Figure 2. One can see in Figure 2a and Figure 2c that, depending on the direction of interparticle interactions, we observe qualitatively different structures. For model **NT111** (Figure 2a) the formation of hexagonal pores is observed. It is interesting that aside from well-defined porous networks, we observe small defects, which are artifacts of the cooling process. These can also be observed very often in experiments.

For model **WT111** (Figure 2c) the formation of a different structure occurs. Also, there is only one aggregate built of almost all molecules in the system. It is surprising that for this case the structure reminds of a honeycomb structure, which is the product of the homotactic polycondensation of 1,3-benzene diboronic acid on a HOPG surface [44]. In addition to these MD results, we have performed l-MC simulations. The results for model **NT111** agree with the MD results. However, the situation is different for **WT111**. The configuration obtained with the l-MC method is clearly different from its MD counterpart. It shows a flower-like structure, instead of a closely packed honeycomb network (Figure 2d). This particular result highlights the problem that the lattice type can enforce the formation of structures that are congruent with its symmetry and not necessarily reproducible by other off-lattice methods. Another aspect that has to be taken into account in such analysis is that even if the methods agree with each other or even with experimental data, the l-MC method idealizes obtained structures due to the limited amount of possible arrangements. This has been already observed by us in the case of tetratopic molecules, which assembled into Kagomé and brickwall networks [39]. In Figure 1b one can see the results for molecule **NL111**, which forms a square-like pattern. It shows that even the directions of interparticle interactions are the same as for **NT111**. The change of the angle θ is a key factor that changes the behavior of molecules of this type.

**Figure 3:**
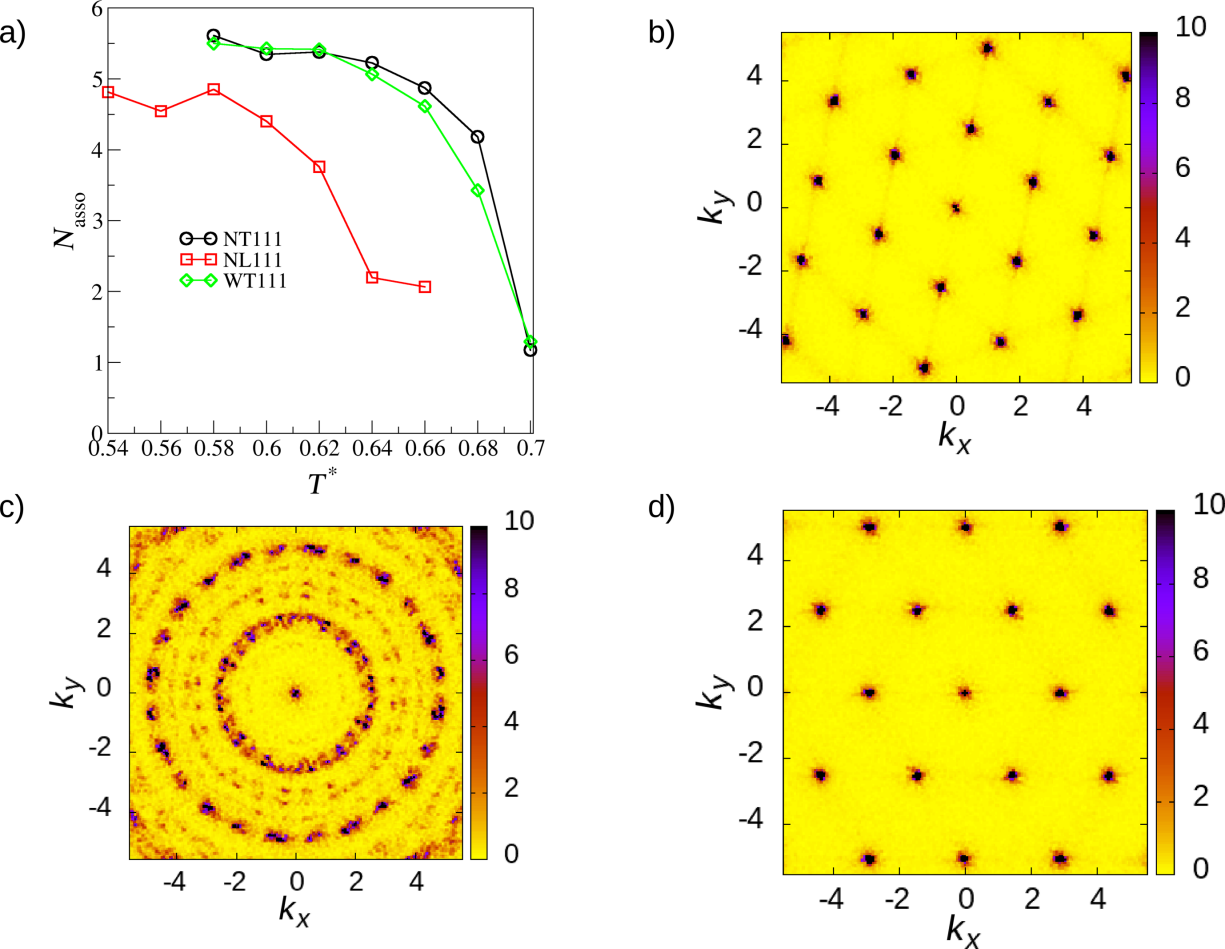
a) Average association number *N*_asso_ as a function of the temperature. b–d) Structure factors calculated for the central segment for the systems **NT111** at T* = 0.58, **NL111** at T* = 0.54, and **WT111** at T* = 0.58, respectively, all with ρ* = 0.2.

To characterize the structures presented in Figure 1 we have computed the average association number, *N*_asso_, and the structure factors with respect to the central segment. *N*_asso_ takes values from 0 to 6, where 0 means that no molecules have interacted and 6 means that all molecules are associated. The structure factor shows the symmetry of the obtained functions. This parameter corresponds to the neutron scattering patterns in the experimental results. The results of our analysis can be found in Figure 3. One can see in Figure 3a that the ordering starts at relatively high temperatures (cf. Figure 2), around T* = 0.62–0.64. Nevertheless, by cooling down the systems we can obtain larger clusters, which is a well-known fact.

In Figure 3b–d diffraction patterns for the systems **NT111**, **NL111**, and **WT111** are shown. It is very interesting that even though the networks of **NT111** and **WT111** are completely different, the structure factor shows hexagonal symmetry for both. In the case of molecule **NL111** we see a diffused diffraction pattern, which means that there are a lot of differently oriented clusters in the system. We have very recently shown that this issue can lead to the wrong interpretation of results [39]. One of the possible solutions to that is to take a fragment of the configuration and to the compute diffraction pattern (or any other orientation-dependent function) from that. Unfortunately, in this particular case, the number of molecules, hence also the number of central segments, was too low to obtain satisfactory statistics.

**Figure 4:**
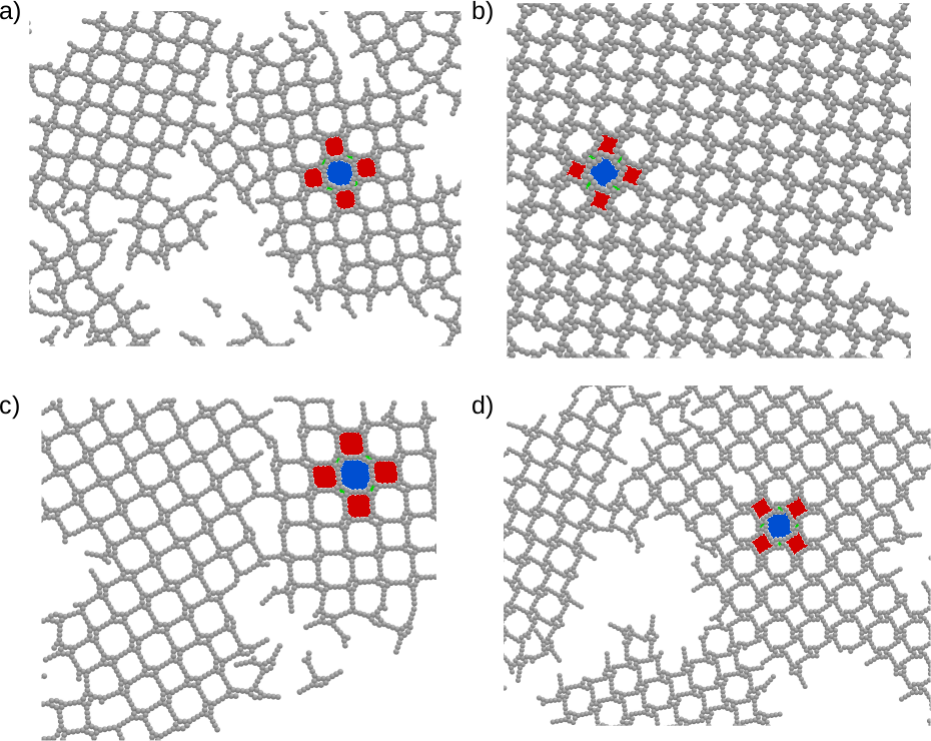
Parts of the configurations of a) **NL221**, b) **WL221** c) **NL321**, and d) **WL321**, all with ρ* = 0.2 at T* = 0.56.

**Figure 5:**
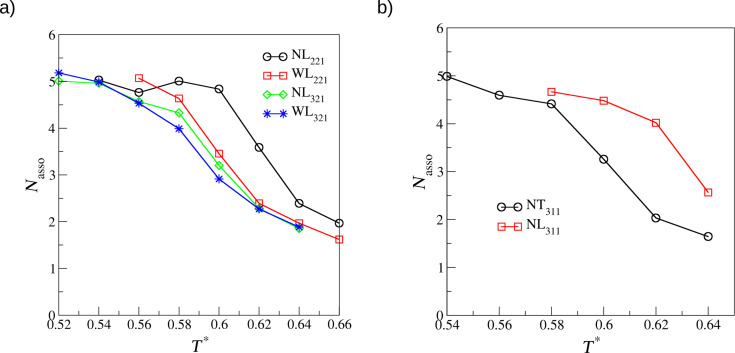
a, b) Average association number, *N*_asso_, as a function of the temperature for the molecules given in the legends.

Let us now proceed to asymmetrical tripod building blocks. Figure 4 shows parts of the configurations of **NL221** (Figure 4a), **WL221** (Figure 4b), **NL321** (Figure 4c), and **WL321** (Figure 4). One can see that for these molecules with θ = 60°all formed networks have square symmetry, similar to that observed in Figure 2b. The only difference between them is due to the direction of the interparticle interactions between the models **NL** and **WL**, which causes a rotation of the square lattice for the latter. It is also interesting that there are three distinguishable pore sizes in every system, marked in color in Figure 4. Structures of this type can be of particular interest for the selective deposition of guest molecules of different size. Another possible application can be found in analytical chemistry and, particularly, in chromatography, where such porous networks can be used as molecular sieves. The fact that there is no distinct difference between the direction of interparticle interactions or the architecture within the molecules with θ = 60°leads us to the conclusion that the key factor for the arrangement of this type of molecules is not the architecture itself but the aforementioned angle between the arms **B** and **C**. To characterize these networks we have again computed the average association number, which can be found in Figure 5a. Again, the ordering transition occurs at relatively high temperatures, that is T* = 0.60–0.62.

**Figure 6:**
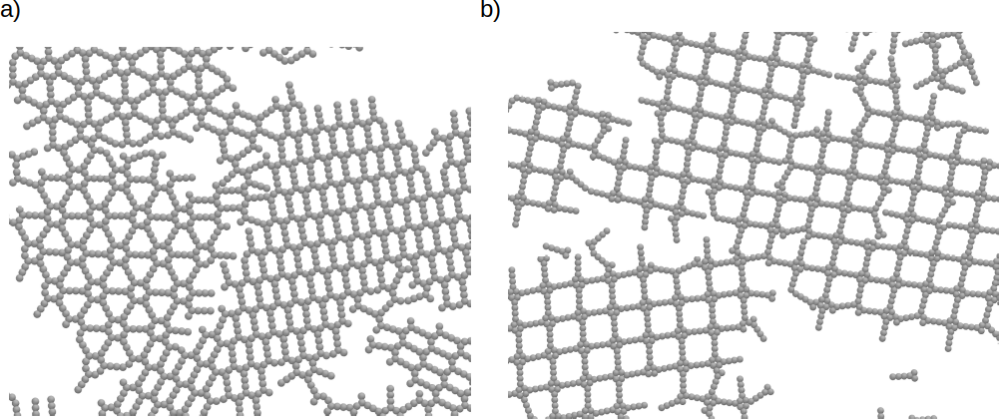
Parts of the configurations for a) **NT311** and b) **NL311**, all with ρ* = 0.2 at T* = 0.58.

The next investigated molecules are **NT311** and **NL311**. Parts of the configurations are shown in Figure 6. **NL311** also forms a square lattice. This result proves our previous conclusion on what is a key factor for the development of these networks. For **NT311** we see a coexistence of two ordered networks, one with parallel structures and one with structures resembling a ship’s-wheel. These results agree with those from the l-MC method. Again, we have characterized these structures and computed the average association number, which can be found in Figure 5b.

## Conclusion

We have shown MD simulation results of the self-assembly of different models of tripod building blocks. We have characterized the self-assembled structures regarding different structural parameters such as theoretical diffraction patterns and average association number. We have found that for molecules with θ = 60° the key factor is the angle itself rather than the direction of interparticle interactions or molecular architecture.

Unfortunately, in the case of tectons with θ = 120°, we can not find a general rule for the prediction of predefined networks. One can see from our simulations that the formed networks highly depend on both the interparticle interactions and the molecular architecture. The sensitivity of the aforementioned variables shows us that simplified approaches are of particular importance because they allow us to examine systems under different conditions more effectively than experiments would do. This is mainly associated to reduced cost and time efforts of the coarse-grained model. Moreover, we have shown that the lattice symmetry in Monte Carlo simulations can enforce the formation of structures, which are not reproducible with off-lattice simulations.

## Acknowledgements

Calculations were carried out at the Academic Computer Centre in Gdansk.
